# One-Pot Terpolymerization of Macrolactones with Limonene Oxide and Phtalic Anhydride to Produce di-Block Semi-Aromatic Polyesters

**DOI:** 10.3390/polym14224911

**Published:** 2022-11-14

**Authors:** Ilaria D’Auria, Sara D’Aniello, Gianluca Viscusi, Elena Lamberti, Giuliana Gorrasi, Mina Mazzeo, Daniela Pappalardo

**Affiliations:** 1 Department of Chemistry and Biology “A. Zambelli”, Università di Salerno, Via Giovanni Paolo II 132, 84084 Fisciano, SA, Italy; 2 Department of Industrial Engineering, Università di Salerno, Via Giovanni Paolo II 132, 84084 Fisciano, SA, Italy; 3 Department of Science and Technology, Università del Sannio, Via de Sanctis snc Benevento, 82100 Benevento, BN, Italy

**Keywords:** block-copolymers, aliphatic polyesters, semi-aromatic polyesters, ROP, ROCOP, macrolactones, limonene oxide

## Abstract

The synthesis of novel block copolymers, namely poly(limonene-phthalate)-*block*-poly(pentadecalactone) and poly(limonene-phthalate)-*block*-poly(pentadecalactone) is here described. To achieve this synthesis, a bimetallic aluminum based complex (**1**) was used as catalyst in the combination of two distinct processes: the ring-opening polymerization (ROP) of macrolactones such as ω-pentadecalactone (PDL) and ω-6-hexadecenlactone (HDL) and the ring-opening copolymerization (ROCOP) of limonene oxide (LO) and phthalic anhydride (PA). The synthesis of di-block polyesters was performed in a *one-pot* procedure, where the semi-aromatic polyester block was firstly formed by ROCOP of LO and PA, followed by the polyethylene like portion produced by ROP of macrolactones (PDL or HDL). The obtained di-block semiaromatic polyesters were characterized by NMR and GPC. The structural organization was analyzed through XRD. Thermal properties were evaluated using differential thermal analysis (DSC) and thermogravimetric measurements (TGA) either in air or in nitrogen atmosphere.

## 1. Introduction

Polyesters are an important class of synthetic polymers, largely used in different field, from packaging to sophisticated biomedical applications [1,2]. Aliphatic polyesters represent the current sustainable alternatives to petroleum-based polymers because of their numerous renewable sources, and facile hydrolytic degradation [3,4].

Aliphatic polyesters can be prepared either by polycondensation of dicarboxylic acids and diols or by *ring opening polymerization* (ROP) of cyclic esters [5,6]. The polycondensation is however difficult to control, and moreover it is difficult to achieve high molecular weight polymer with this process [7]. On the other side, the ROP of lactones and lactides, promoted by metal-based systems, organic catalysts or enzymes, appeared the best route for the preparation of high molecular weight polyesters with good control on the composition, molecular weight, microstructure [2]. The ROP of cyclic esters, especially if it is promoted by metal-based catalysts, is the best method to improve structural control and, in turn, for modulation of the polymers thermo-mechanical and degradation properties. Several metal-based catalysts have been developed, able to promote the ROP of homo- and co-polymerization of diverse lactones and lactides, also with control on the polymer microstructure and distribution of the comonomer along the polymeric chain. In particular aluminum bis-phenoxyimine based catalysts have been largely used as ROP catalysts, as well as the closely related aluminum phenoxyimine catalyst [8,9].

One drawback of the ROP is, however, the scarce availability of suitable monomers. On the contrary the closely related *ring opening copolymerization* (ROCOP) of o-carboxyanhydrides and epoxides may take advantages from the variety of side-chain functionalities of o-carboxyanhydrides and epoxides, resulting in polyester formation having a variety of structural features and to polyesters that cannot be prepared directly from the ROP of lactones [10,11]. In particular semi-aromatic polyesters, not accessible by ROP, have been obtained via ROCOP, which represents a possible way to increase backbone “rigidity” through the incorporation of aromatic or strained heterocyclic units.

Copolymerization represents a versatile method to modulate, modify and improve the polymer properties [11]. By properly selecting the comonomer (and its origin petro-based or bio-based), either complete or partly bio-based copolymers can be obtained. By different copolymerization methods, polymer chemists have had access to a large variety of copolymers, with different microstructure, from random, to block, to multiblock. In particular, the *living* ring-opening copolymerization method, by sequential addition of different monomers, provided precise block copolymers [12]. Recently, the combination of ROP and ROCOP has allowed the preparation of di-block polyesters starting from a *one-pot* mixture of o-carboxyanhydrides and epoxide and cyclic esters in “switchable” catalysis method between ROP and ROCOP [10]. Williams et al. in 2014 pioneering this novel catalytic approach, achieved by a chemoselective control, where the switch of the polymerization mechanism was dictated by the depletion of a specific monomer and by the chemistry of the metal–polymer chain end group [13]. Within this approach, diverse catalytic systems, [14,15] including multinuclear organometallic catalysts [16,17] have been described. In this regards we recently described a monometallic phenoxy-imine aluminum complex and a bimetallic *salen*–type aluminum complex in the ROP of diverse macrolactones (i.e., lactones having more than 14 atoms) and in the ROCOP of phthalic anhydride (PA) with cyclohexene oxide (CHO). By combination of the two distinct processes in a *one pot* procedure, diblock semiaromatic polyesters were prepared [18]. In details, the used macrolactones were ω-pentadecalactone (PDL), which homopolymer, namely the PPDL, may represents the sustainable alternative to linear low density polyethylene [19], and the ω-hexadecenlactone (HDL), an unsaturated version of the macrolactones, which could be used to further functionalize the polymers thereof [20,21]. In our approach, the semi-aromatic polyester block was formed first, followed by the ROP of macrolactones producing a *polyethylene-like* second block [18].

In the search for monomers derived from renewable resources [22], limonene oxide (LO) is receiving increasing attention. LO is indeed obtained from limonene, a bio-based and non-food resource, mainly derived from the peel of citrus fruits as side product of the orange industry. In 2004, Coates et al. reported the copolymerization of LO and CO_2_ in the presence of a β−diiminate zinc catalyst to form poly(limonene carbonate) [23]. This result was followed by a great variety of polymers which have been derived from limonene [24]. Just to mention a few, the modification of poly(limonene carbonate) using bio-based cyclic ester ε-decalactone as a comonomer to give ABA thermoplastic elastomers, obtained by sequential polymerization [25], or the block copolymer poly(limonene carbonate)-block-poly(lactide) obtained by one pot simultaneous copolymerization of LO/CO_2_ with lactide [26]. We also previously have used dinuclear aluminum complexes bearing dinaphthalene bridged Schiff bases ligands in the ROCOP of PA with cyclohexene oxide CHO and limonene oxide LO [27]. In continuation of our studies, we have now extended the approach to the preparation of semi-aromatic di-block polyesters, having one block derived from the ROCOP of LO and PA, and a second block *polyethylene-like*, originated by the ROP of macrolactones. Herein the one pot synthesis of the novel copolymers is reported, along with the detailed microstructural and thermal characterization. 

## 2. Materials and Methods

All manipulations of moisture and air-sensitive chemicals were performed under an inert atmosphere using standard Schlenk techniques or in a MBraun Labmaster glove box. Glassware and vials used in the polymerization were dried in an oven at 120 °C overnight and exposed three times to vacuum-nitrogen cycles. Monomers (Sigma-Aldrich) were purified prior to use: ω-6-hexadecenlactone (6-HDL), ω-pentadecalactone (PDL) and limonene oxide (LO) were distilled under vacuum on CaH_2_ and stored over 4Å molecular sieves. Phthalic anhydride (PA) was crystallized from dry toluene. The aluminum complex was synthesized according to previously reported procedures [15,28]. Toluene was refluxed over Na and distilled under nitrogen. CDCl_3_ was purchased from Eurisotop and used as received. The bis(triphenylphosphine)iminium chloride salt (PPNCl) and all other reagents and solvents were purchased from Sigma-Aldrich and used as received unless stated otherwise.

### 2.1. Material Characterization

*NMR analysis*. The NMR spectra of polymers were recorded in CDCl_3_ at room temperature on Bruker Advance 300, 400, 600 MHz spectrometers [^1^H: 300.13, 400.13, 600.13 MHz; ^13^C: 75.47, 100.62, 150.92 MHz, respectively]. The resonances are reported in ppm (δ) and the coupling constants in Hz (J) and are referenced to the residual solvent proton at δ = 7.26 ppm. ^13^C NMR spectra are referenced to ^13^C signal of CDCl_3_ at δ = 77.16 ppm. Spectra were recorded on Bruker TopSpin v2.1 software (Bruker, Billerica, MA, USA). Data processing was performed using TopSpin v2.1 software.

The ^2^D DOSY spectra of the di-block copolymers were recorded on a Bruker Avance 600 spectrometer: 2 mg of the polymer were dissolved in 0.5 mL of CDCl_3_ and the spectra were recorded at room temperature without spinning; the parameters δ and Δ were kept constant during the experiments, whereas G was varied from 5 to 95% in 25 steps, 64 scans per step.

*Gel permeation chromatography measurements (GPC)*. Molecular weights (M_n_ and M_w_) and dispersities (*Ð*) of polymeric samples were measured by size exclusion chromatography. The measurements were performed at 35 °C on an Agilent 1260 Infinity II HTGPC (RI) system equipped with a system of two PLGEL column 5 μm MIXED-C (7.5 × 300 mm). Tetrahydrofuran was used as eluent at a flow rate of 1.0 mL min^−1^. Narrow polystyrene standards were used as reference, and data processing was performed using Agilent GPC/SEC Software A.02.02 (Agilent Technologies, Palo Alto, CA, USA).

*X-ray diffraction (XRD) analysis.* XRD patterns were obtained in reflection mode with an automatic Bruker diffractometer (equipped with a continuous scan attachment and a proportional counter), using nickel-filtered Cu Kα radiation (Kα = 1.54050 Å) and operating at 40 kV and 40 mA, step scan 0.05° of 2ϑ and 3s of counting time.

*Thermo Gravimetric Analyses (TGA).* TGA were carried out with a Mettler TC-10 thermobalance (Mettler-Toledo GmbH, Greifensee, Switzerland) from 25 to 700 °C at a heating rate of 10 °C/min under air and nitrogen flow.

*Differential scanning calorimetry (DSC)*. DSC was used to evaluate thermal transitions of the samples. The used apparatus was a DTA Mettler Toledo (DSC 30, Mettler Toledo, Greifensee, Switzerland) under a nitrogen atmosphere. The samples were characterized in a dynamic test from −50 °C to 200 °C at a heating rate of 10 °C/min.

### 2.2. Material Synthesis

#### 2.2.1. Synthesis of Poly(limonene-phthalate)-block-poly(hexadecenlactone)

A typical polymerization is described herein for the sample of run 1 in Table 1. The polymerization of run 2 was carried out analogously, but the time was 2 days. A Schlenk tube was charged sequentially with phthalic anhydride (118.48 mg, 0.8 mmol), limonene oxide (152.23 mg, 1.0 mmol), ω-6-hexadecenlactone (252.39 mg, 1.0 mmol), complex **1** (3.94 mg, 10 μmol), and PPNCl (11.5 mg, 20 μmol) in 0.5 mL of toluene. The mixture was thermostated at 130 °C and magnetically stirred for required time, and then cooled to room temperature. The mixture was added dropwise to methanol under rapid stirring. The precipitated polymer was recovered by filtration, washed with methanol and dried at 30 °C overnight in a vacuum oven. ^1^H NMR (600 MHz, CDCl_3_, 298 K): 1.25 (14H, CH_2_(*CH*_2_)_7_CH_2_); 1.75–1.55 (4H, C(O)CH_2_C*H*_2_ and C*H*_2_CH_2_O); 2.26 (2H, *CH*_2_C=O); 4.04 (2H, -*CH*_2_O); 4.65 (2H, C*H*_2_=); 5.37(2H, -C(*H*)v); 5.51 (1H, OC*H*CH_2_); 7.51, 7.70 (4H, Ar).

#### 2.2.2. Synthesis of Poly(limonene-phthalate)-block-poly(pentadecalactone)

A typical polymerization is described herein for the sample of run 3 in Table 1. The polymerization of run 4 and 5 were carried out analogously, but the conditions were different, as specified in Table 1. A Schlenk tube was charged sequentially with phthalic anhydride (118.48 mg, 0.8 mmol), limonene oxide (152.23 mg, 1.0 mmol), ω-pentadecalactone (240.38 mg, 1.0 mmol), complex **1** (3.94 mg, 10 μmol), and PPNCl (11.5 mg, 20 μmol) in 0.5 mL of toluene. The mixture was thermostated at 130 °C and magnetically stirred for required time, and then cooled to room temperature. The mixture was added dropwise to methanol under rapid stirring. The precipitated polymer was recovered by filtration, washed with methanol and dried at 30 °C overnight in a vacuum oven. ^1^H NMR (600 MHz, CDCl_3_, 298 K): 1.25 (20H, CH_2_(*CH*_2_)_10_CH_2_); 1.59 (4H, *CH*_2_(CH_2_)_10_*CH*_2_); 2.26 (2H, *CH*_2_C=O); 4.03 (2H, -*CH*_2_O); 4.69 (2H, C*H*_2_=); 5.51 (1H, OC*H*CH_2_); 7.51, 7.70 (4H, Ar).

## 3. Results and Discussion

### 3.1. Synthesis of di-Block Polyesters

**Scheme 1:**
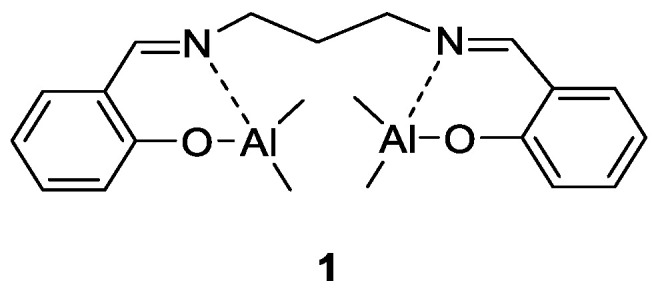
Structure of the bimetallic aluminum complex (**1**) used in this work.

The synthesis of the block copolymers was carried out in the presence of a bimetallic aluminum complex (**1**), depicted in Scheme 1, in which each aluminum atom is coordinated to a phenoxy-imine moiety; the two phenoxy-imine fragments are bounded each other via a backbone of three carbon atoms. The proximity between the two aluminum can allow cooperation phenomena between the aluminum reactive centers. Complex **1** has been selected since it was already described as an effective catalyst not only in the ROP of several macrolactones such as ω-pentadecalactone (PDL), ethylene brassilate (EB) and ω-hexadecenlactone (HDL) but also in the ROCOP of PA with CHO, as well as in the combination of these two catalysis [18]. Previous studies demonstrated that, in the ROP of cyclic esters, the bimetallic complex **1** was more active than the closely related monometallic one because of positive cooperative effects between the two metal centers. Differently, in the ROCOP no difference in terms of activity was observed between the two catalysts.

**Scheme 2:**
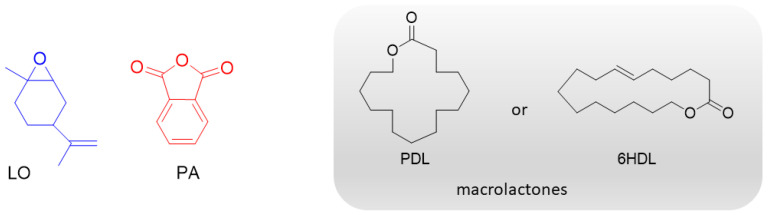
Structures of the monomers used in the copolymerization.

**Scheme 3:**
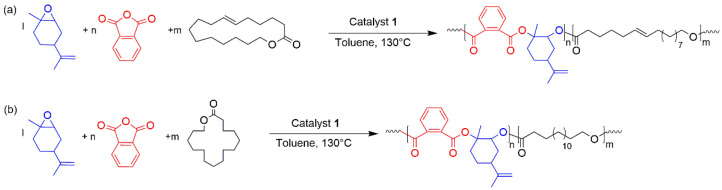
(**a**) *One-pot* terpolymerization of HDL with LO and PA (**b**) *One-pot* terpolymerization of PDL with LO and PA.

In this work we extended these studies and tested complex **1** in the combined catalysis ROCOP/ROP for a novel combination of monomers; in details of phthalic anhydride (PA), limonene oxide and macrolactones such as ω-pentadecalactone (PDL), and the ω-hexadecenlactone (HDL) were used (Scheme 2). The reactions were carried out at 130 °C to force the reactivity of limonene oxide. Moreover, a higher amount of limonene oxide in comparison to PA was used to favor the insertion of the macrolactone on the alkoxylate-aluminum bond because the carboxylate intermediate is unable to initiate the ROP of the cyclic lactone. The synthesis of the block copolymers was carried out by a *one pot* procedure (Scheme 3). The produced polymers were characterized by ^1^H, ^13^C and DOSY NMR, GPC and MALDI-ToF-MS analyses. Representative polymerization data are summarized in Table 1.

**Figure 1:**
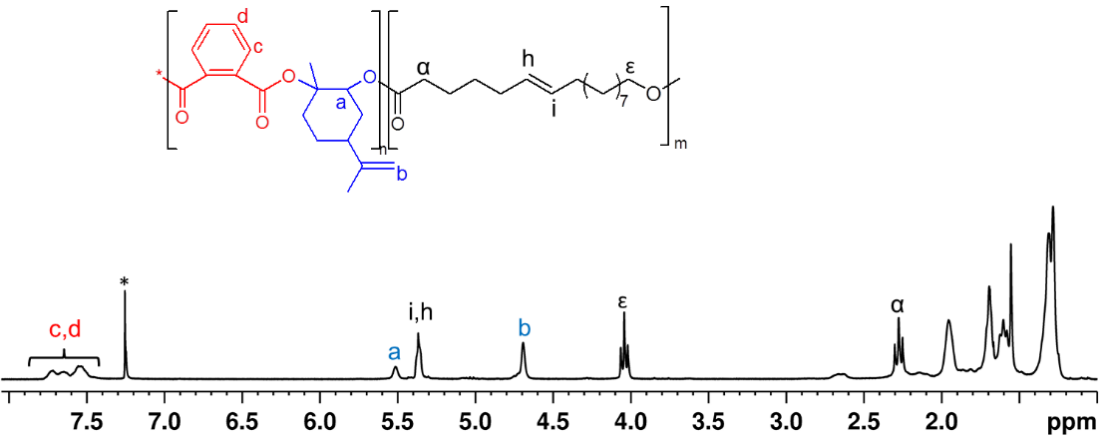
^1^HNMR (600 MHz, CDCl_3_, RT) of poly(limonene-phthalate)-*block*-poly(hexadecenlactone).

The co-monomers, LO and PA and the macrolactone (either PDL or HDL), were added simultaneously in the polymerization medium and the reaction was monitored by ^1^H NMR analysis of minimal aliquots of the polymerization mixture. The copolymer composition was estimated by ^1^H NMR analysis performed in CDCl_3_ by using a 600 MHz spectrometer, by comparing the integrals of the signals of epoxide/anhydride sequences with those of macrolactone homosequences. The monitoring of terpolymerization promoted by complex **1** showed that after 2 h the phthalic anhydride was completely consumed while no conversion of HDL was detected (run 1, Table 1). After 48 h the conversion of about 50% of macrolactone was reached. The ^1^H NMR spectrum of the crude polymer accounted for a copolymer containing a 1:1:0.6 ratio of the monomers in agreement with the conversions monitored during the reaction. To increase the conversion of the macrolactone, and the length of the poly(hexadecenlactone) fragment, an analogous terpolymerization was performed for prolonged time; in this case the almost complete conversion of HDL was achieved after four days (run 2, Table 1). The ^1^H NMR spectrum of the obtained polymer showed all the characteristic signals of the semiaromatic block and of the pentadecenlactone block (Figure 1). The two blocks of the poly(limonene-phthalate)-*block*-poly(hexadecenlactone) had almost the same length.

The di-block structure of the copolymer was assessed by different experiments. As a first confirmation of this hypothesis, the GPC analysis showed always monomodal distributions of the molecular masses. As a general behavior, the copolymer molecular weights values obtained by GPC were lower than the theoretically ones, calculated on the basis of the conversion of the monomers (around 43 KDa). Notably, these copolymers are novel, therefore the calibration curves are not available for these materials. In addition to this, it is worth to note that the radius of gyration of the copolymers is highly dependent on the chemical composition, and thus the molecular weight obtained by GPC should be considered with special care. Moreover, the adventitious presence of protic impurities, commonly present especially in the monomers, may act as chain transfer agents, thus enlarging the molecular weight distribution and being responsible of the poor control in the molecular weight. This is quite a common feature in the case of ROCOP of epoxides and anhydrides. The GPC data, however gives important indication on the molecular weight distribution, which is monomodal in all cases, thus demonstrating that block copolymers have been produced (and not just mixture of two polymers).

**Figure 2:**
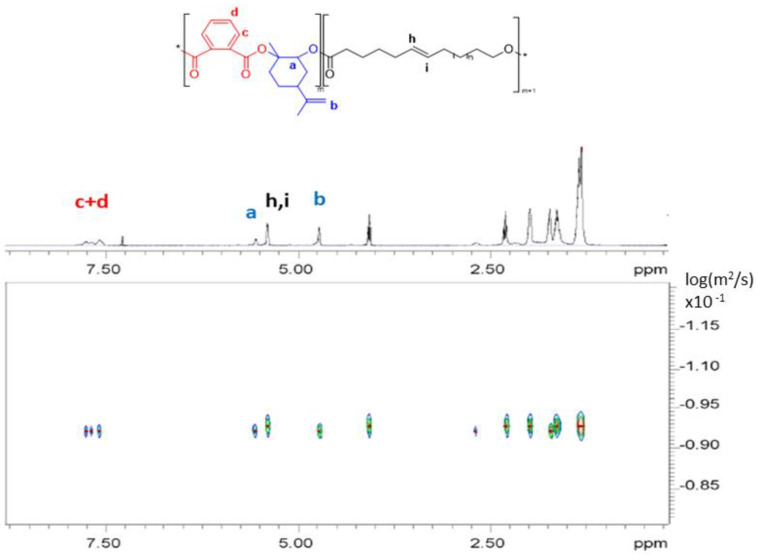
^2^ D DOSY NMR (600 MHz, CDCl_3_, RT) of poly(limonene-phthalate)-*block*-poly(hexadecenlactone).

To further and definitely support the di-block structure, and to rule out that a mechanical mixture of the two polymers (i.e., polylimonene-phthalate *and* polyhexadecenlactone) was instead obtained, a DOSY NMR analysis was carried out on the sample. The DOSY spectrum (see Figure 2) showed that the signals related to the two blocks of the polyesters had the same diffusion coefficient, thus confirming the production of a di-block copolymer poly(limonene phthalate)-*block*-poly(hexadecenlactone).

**Figure 3:**
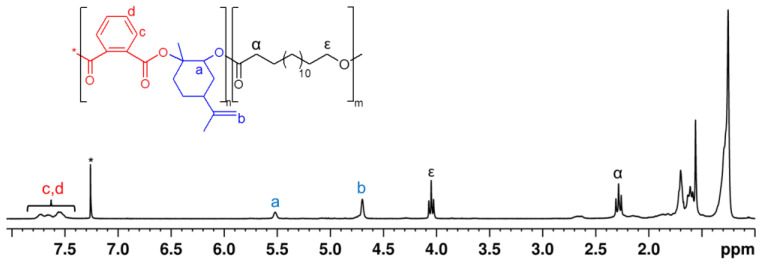
^1^HNMR (600 MHz, CDCl_3_, RT) of poly(limonene-phthalate)-*block*-poly(pentadecalactone). The signals in the spectrum have been assigned to the proper hydrogen, as indicated in the structure.

As expected, a lower reactivity was observed with the more refractarium ω-pentadecalactone; in this case after two days a conversion of 45 % was achieved (run 3, Table 1), while five days were necessary to obtain a quantitative conversion of this co-monomer (run 4, Table 1). The ^1^H NMR spectrum of this block copolymers is shown in Figure 3; the characteristic signals of the semiaromatic block and of the pentadecalactone block have been evidenced.

### 3.2. X-ray Diffraction Analysis of Copolymers

**Figure 4:**
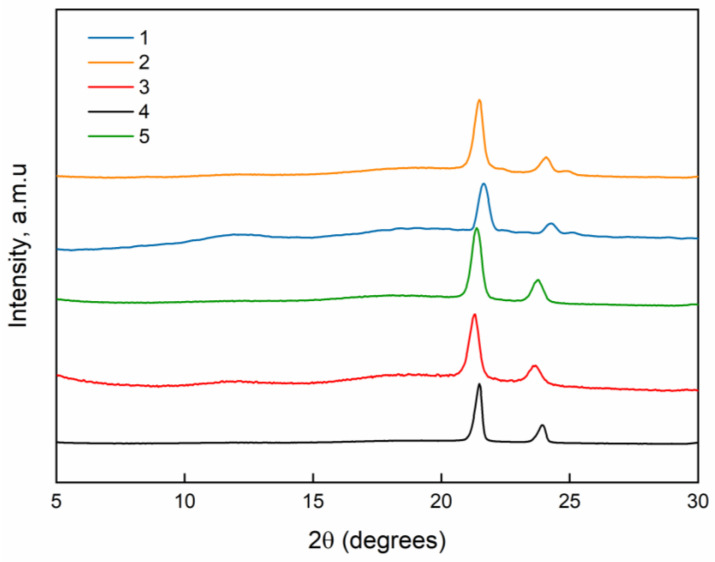
XRD spectra of the samples reported in Table 1.

Figure 4 reports the XRD diffraction patterns of all the synthetized samples. It is evident that all the copolymers showed a similar polyethylene-like structure. The main peaks, for all the samples, range between 21.6° and 24.5° of 2 ϑ for sample 1 and 21.3° and 23.3° of 2 ϑ for sample 3. The peak around 25° of 2 ϑ is typical of orthorombic form of PE, that corresponds to (200) reflection [29]. The similarity of XRD spectra of aliphatic long-chain polyesters and those of polyethylene (PE) has been already reported for materials obtained from saturated and unsaturated macrolactones [30], Blocks derived from copolymerization of PA and LO are supposed to be parts of the amorphous phase, and then not detectable with sharp diffraction angles. From the diffractograms it is possible to derive the fraction of crystalline phase, dividing the area of the crystalline peaks by the total area. The diffractogram of the amorphous phase was drawn over the baseline, with its maximum at around 20 of 2 ϑ [31]. Samples 1 and 2 show degree of crystallinity of around 55%while samples 3, 4, 5 show similar degrees of crystallinity (around 70%).

### 3.3. Thermal Analysis

Differential thermal analysis (see Supporting Information) allowed to evaluate the melting temperatures for all the synthetized samples. Table 2 reports the experimental data. It is evident that the LO block does not influence the melting temperature of the ter-polymers. The PDL blocks impart to the materials higher melting temperatures than the materials with HDL. Samples 1 and 2 have the same melting temperature, as well as samples 3, 4, 5. The lower degree of crystallinity evaluated by XRD spectra (Section 3.2) for samples 1 and 2 is a key to interpret such behavior. The lower is the crystallinity, the lower is the melting point.

**Figure 5:**
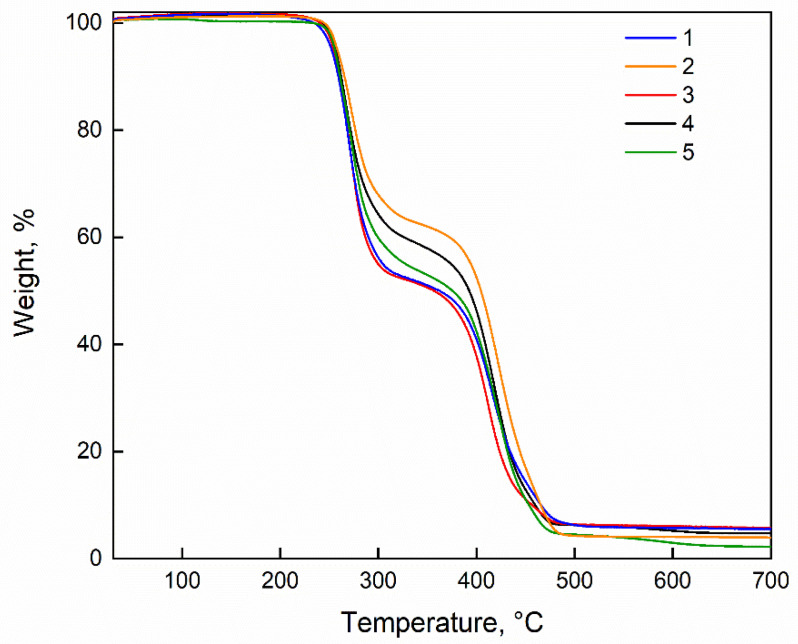
Thermogravimetric curves for all the samples in nitrogen atmosphere.

**Figure 6:**
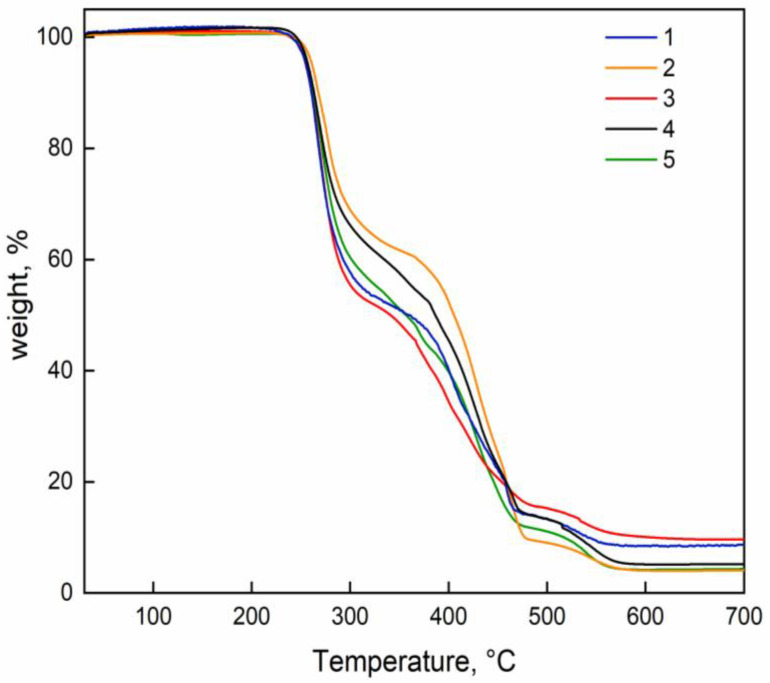
Thermogravimetric curves for all the samples in air atmosphere.

Thermogravimetric curves (Figure 5 and Figure 6) allowed to evaluate the degradation behavior for all analyzed samples.

It is evident that the more is the homopolymer conversion the higher is the degradation temperature. As previously reported [32] the thermal degradation of HDL polymers is a very complex phenomenon. In all cases analyzed, it is reasonably to hypothesize that the first degradation step is due to the LO/PA blocks. The second degradation step in nitrogen can be mainly ascribed to the production of 5-hexenoic acid, CO_2_, H_2_O, and methyl pentanoate. In oxygen environment the additional two degradation steps can be attributed to short chain carboxylic acids formation and production of CO_2_, CO, H_2_O [33].

## 4. Conclusions

The catalytic behavior of a bimetallic aluminum complex was explored in the *one-pot* copolymerization of LO, PA and a macrolactone (PDL or HDL). The catalyst nicely dictated the selectivity of the monomer insertion, giving rise to two sequential polymerizations: first the alternate copolymerization of LO and PA, followed by the homopolymerization of the macrolactone (PDL and HDL). The formation of the block-copolymers was confirmed by NMR, GPC and ^2^D DOSY NMR analyses. The obtained copolymers were also characterized by DSC, TGA and RX analyses.

The obtained poly[(limonene-phthalate)-*block*-poly(pentadecalactone) and poly[(limonene-phthalate)-*block*-poly(hexadecenlactone) are novel; to the best of our knowledge this is the first time in the literature that such combination of comonomers is used in a *one-pot* ROP and ROCOP procedure, to produce this kind of semi-aromatic block copolymers. These materials are novel, and can have several potential applications in various field. To mention a few, as compatibilizer of polymeric blends made of aliphatic and aromatic components, or for more sophisticated uses, where block copolymers represent ideal nanoscale tools, due to their peculiarity of self-assembling into nanometer-sized structures.

## Figures and Tables

**Table 1:** Ter-polymerizations of LO, PA and macrolactones promoted by **1** ^a^.

Run	PDL ^d^ (eq)	HDL ^d^ (eq)	LO ^d^ (eq)	PA ^d^ (eq)	t (days)	^b^ Conv (%)	^c^ M_nGPC_ (KDa)	^c^ *Ð*
1	-	100	100	80	2	HDL = 54	4.7	1.80
2	-	100	100	80	4	HDL = 95	5.4	2.18
3	100	-	100	80	2	PDL = 45	6.5	1.32
4	100	-	100	80	5	PDL > 99	4.8	1.23
5	200	-	200	150	3	PDL = 42	5.1	1.47

^a^ All reactions were carried out at 130 °C with **1** = 10 μmol in toluene (0.5 mL), PPNCl = 20 μmol. ^b^ Conversions of PDL or HDL were determined by ^1^H NMR; PA conversions were always >99%. ^c^ GPC data: M_nGPC_ = number average molecular weights; *Ð* = dispersities. ^d^ Equivalent of the monomer in the polymerization feed.

**Table 2:** Melting temperatures of all samples evaluated by DSC.

Sample	T_m_ (°C)
1	65
2	63
3	82
4	85
5	84

## Data Availability

The data presented in this study are available on request from the corresponding authors (D.P. and M.M.).

## Supplementary Materials

The following supporting information can be downloaded at: https://www.mdpi.com/article/10.3390/polym14224911/s1, Figure S1: DSC spectra of all the samples.
