# Supplementary material for: One-Pot Terpolymerization of Macrolactones with Limonene Oxide and Phtalic Anhydride to Produce di-Block Semi-Aromatic Polyesters
Source: Polymers (Basel). 2022 Nov 14;14(22):4911. doi: 10.3390/polym14224911 (PMC9695062; doi:10.3390/polym14224911)
Supplement: Supplementary file 1 [file polymers-14-04911-s001.zip › polymers-1993032-supplementary.pdf]

Supporting Information

One-Pot Terpolymerization of Macrolactones with Limonene Oxide and Phtalic Anhydride to Produce di-Block Semi-Aromatic Polyesters

Ilaria D'Auria [1,†], Sara D'Aniello [1,†], Gianluca Viscusi [2], Elena Lamberti [2], Giuliana Gorrasi [2], Mina Mazzeo [1,*] and Daniela Pappalardo [3,*]

[1] Department of Chemistry and Biology "A. Zambelli", Università di Salerno, Via Giovanni Paolo II 132, 84084 Fisciano, SA, Italy
[2] Department of Industrial Engineering , Università di Salerno, Via Giovanni Paolo II 132, 84084 Fisciano, SA, Italy
[3] Department od Science and Tecnology, Università del Sannio, Via de Sanctis snc Benevento, BN, Italy
* Correspondence: mmazzeo@unisa.it (M.M.); pappalardo@unisannio.it (D.P.)
† These authors contributed equally to this work.

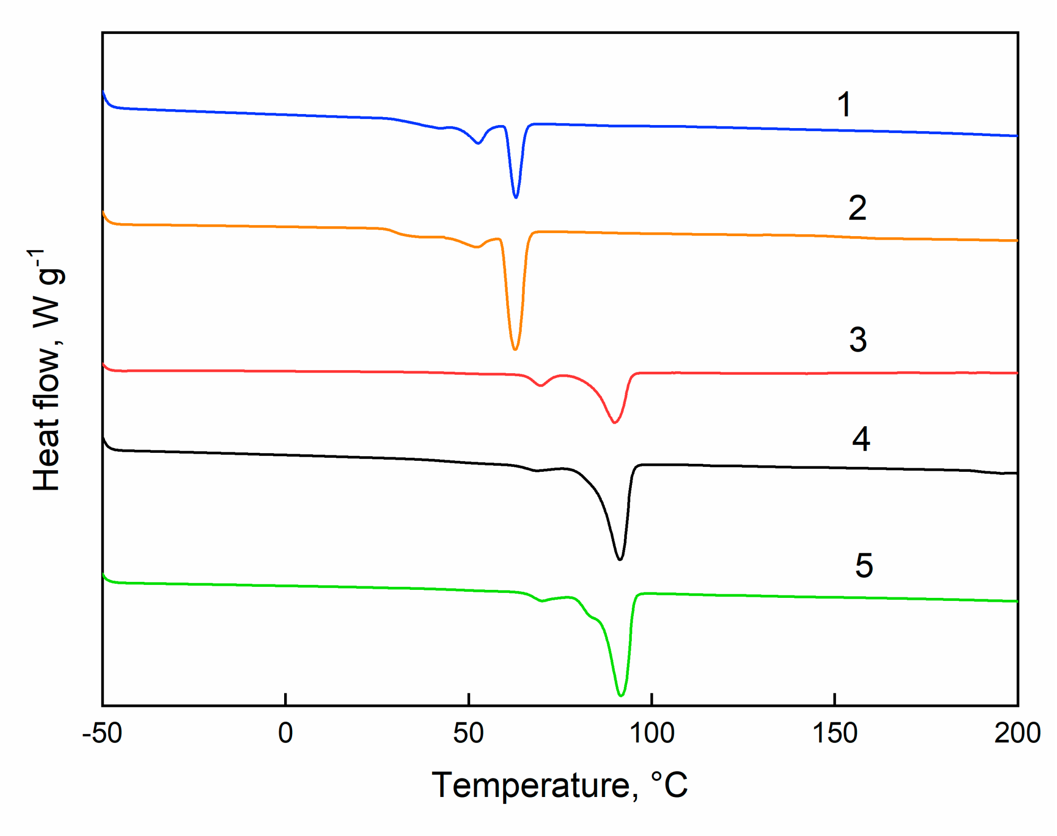

Figure S1. DSC spectra of all the samples.
